# Advances in Medications and Tailoring Treatment for Alcohol Use Disorder

**DOI:** 10.35946/arcr.v37.1.02

**Published:** 2015

**Authors:** Chamindi Seneviratne, Bankole A. Johnson

**Affiliations:** Chamindi Seneviratne, M.D., is an assistant professor in the Department of Psychiatry, Institute for Genome Sciences; Bankole A. Johnson, D.Sc., M.D., MBChB, M.Phil., FRCPsych, DFAPA, FACFEI, is the Dr. Irving J. Taylor Professor and chair in the Department of Psychiatry, professor of pharmacology and professor of anatomy and neurobiology, and director of the Brain Science Research Consortium Unit, both at the University of Maryland School of Medicine, Baltimore, Maryland.

**Keywords:** Alcohol use disorder, alcoholism, brain disorder, medications, treatment, environmental factors, biological factors, genetic factors, pathophysicological mechanisms, molecular mechanisms, genetic polymorphisms, pharmacogenetics

## Abstract

Alcohol use disorder (AUD) is a chronic heritable brain disorder with a variable clinical presentation. This variability, or heterogeneity, in clinical presentation suggests complex interactions between environmental and biological factors, resulting in several underlying pathophysiological mechanisms in the development and progression of AUD. Classifying AUD into subgroups of common clinical or pathological characteristics would ease the complexity of teasing apart underlying molecular mechanisms. Genetic association analyses have revealed several polymorphisms—small differences in DNA—that increase a person’s vulnerability to develop AUD and other alcohol-related intermediate characteristics, such as severity of drinking, age of AUD onset, or measures of craving. They also have identified polymorphisms associated with reduced drinking. Researchers have begun utilizing these genetic polymorphisms to identify alcoholics who might respond best to various treatments, thereby enhancing the effectiveness of currently tested medications for treating AUD. This review compares the efficacy of medications tested for treatment of AUD with and without incorporating genetics. It then discusses advances in pre-clinical genetic and genomic studies that potentially could be adapted to clinical trials to improve treatment efficacy. Although a pharmacogenetic approach is promising, it is relatively new and will need to overcome many challenges, including inadequate scientific knowledge and social and logistic constraints, to be utilized in clinical practice.

Despite decades of research on various methods for treating alcohol use disorder (AUD), AUD remains prevalent throughout the world, making it critical to develop a more comprehensive approach to address the issue. Heavy drinking is the third largest risk factor for global disease burden, leading to enormous social and economic decline (World Health Organization 2014). Each year, alcohol misuse is attributed to approximately 88,000 deaths in the United States and 2.5 million deaths worldwide (Centers for Disease Control and Prevention 2014). Many individuals who drink are able to consume small amounts of alcohol without progressing into heavy drinking that often leads to AUD. However, in the United States alone, approximately 13 percent of those who drink meet criteria for AUD (Friedmann 2013). Despite community education programs on the consequences of harmful drinking, only about 15 percent of those who have an AUD seek treatment, citing reasons that include social stigma, expense, skepticism about treatment efficacy, lack of knowledge on available treatment options, and lack of treatment facilities (National Institute of Alcohol Abuse and Alcoholism 2014).

Finding treatments that successfully help people regulate their drinking or stop drinking altogether is a primary goal of AUD treatment researchers. Along with psychosocial treatments, researchers have been developing and testing pharmaceuticals that can help people with AUD reach their treatment goals. To date, multiple compounds have been tested in pre-clinical studies and phase ll clinical trials. However, the U.S. Food and Drug Administration (FDA) has only approved three specifically for treating AUD (Litten et al. 2014): oral and long-acting injectable naltrexone, acamprosate, and disulfiram. Some European countries have approved nalmefene and sodium oxybate for AUD treatment. Several other drugs, including ondansetron, topiramate and gabapentin, which are drugs approved to treat nausea (ondansetron) and seizures (topiramate and gabapentin), also have shown promise for treating AUD (Johnson et al. 2003; Mason et al. 2012, 2014; Sellers et al. 1994). All of these medications, except disulfiram (see textbox), modulate the neuronal pathways governing the urge or propensity to drink, withdrawal-related symptoms, or maintaining abstinence.

Although naltrexone and acamprosate are used to treat patients, they have not shown strong effects in achieving abstinence or non–heavy-drinking levels in phase II clinical trials (Cochrane Primary Care 2013*a*,*b*). In an effort to develop more effective medications, researchers increasingly are focusing on two goals: (1) improving the efficacy of existing medications and (2) discovering new drug targets. To improve the efficacy of existing medications, researchers are trying to identify subgroups of AUD patients with common underlying pathophysiology who are more likely to respond to certain medications. Such an approach would control for physiological and environmental variations that play a major role in people’s vulnerability to AUD and their response to medication. The challenge is finding ways to specifically and accurately identify subgroups. For example, clinical presentation can vary widely, and there is little consensus as to what constructs should be used to delineate subgroups (Johnson 2010). Genetics holds more promise. The traits that encompass the *Diagnostic and Statistical Manual of Mental Disorders, Fourth Edition* (DSM–IV) diagnosis of alcohol addiction and misuse are highly heritable (Goldman et al. 2005), with some but not all of the seven DSM–IV diagnostic criteria having a genetic predisposition (Kendler et al. 2012). In addition, genetic association analyses suggest that several clinical subtypes, including age of onset of problem drinking, severity of drinking, patterns of drinking, alcohol withdrawal, and other comorbid psychiatric conditions, share specific genetic differences, known as polymorphisms. Therefore, employing these clinical subtypes that are intermediate to disease diagnosis, and the genes associated with the disease, seem to be a more plausible and comprehensive approach to identifying treatment responders. Perhaps focusing on the diagnostic criteria that are controlled by genetic factors will afford greater statistical power to mine underlying genetic factors associated with AUD pathophysiology. Additionally, the genetic variations in genes encoding enzymes that determine the bioavailability of a medication, receptor binding and uptake sites, and enzymes involved in a medication’s elimination also could determine individual’s variable responses to medications. This article will first present an overview of recent findings in AUD pharmacogenetic research, followed by a discussion on how preclinical genetic research can be adopted to improve the current status of the pharmacogenetics of AUD.

**Disulfiram**

Disulfiram (Antabuse) was the first medication available for the treatment of alcohol use disorder, and it remains the most widely prescribed medication in some countries. Disulfiram inhibits the low Km alcohol metabolism enzyme aldehyde dehydrogenase 2 (ALDH2) in the liver and the brain, increasing the downstream acetaldehyde levels (Vasiliou et al. 1986). If a person taking disulfiram drinks alcohol, the resulting acetaldehyde levels cause an aversive reaction that is characterized by nausea, vomiting, headaches, a flushed face and neck, and sometimes rare symptoms that include vertigo, blurred vision, hypotension, and syncope (McMahon 1980). Because of this very unpleasant experience, patients often lack motivation to remain compliant. In the United States, disulfiram is rarely prescribed because of its potentially serious side effects.

In the brain, where catalase is the primary ethanol-metabolizing enzyme, ALDH2 is expressed in very low levels. Acetaldehyde produced in the ventral tegmental area (VTA) of the brain by catalase was shown to be rewarding (Karahanian et al. 2011), but it is not clear whether disulfiram affects acetaldehyde levels generated via catalase. Recent evidence from various groups also has demonstrated that disulfiram’s mechanism of action is more complex and, in addition to ALDH, may target other proteins such as dopamine catabolizing enzymes, particularly, dopamine beta-hydroxylase (Gaval-Cruz et al. 2008; McCance-Katz et al. 1998). Furthermore, the primary metabolite of disulfiram, diethyl- dithiocarbamate, is active and has many protein targets, including transcription factor nuclear factor kappa-B (NF-κB) that can impact many neurotransmitter systems simultaneously. To date, there have been no pharmacogenetic studies conducted using disulfiram.

ReferencesGaval-CruzMSchroederJPLilesLCEffects of disulfiram and dopamine beta-hydroxylase knockout on cocaine-induced seizuresPharmacology, Biochemistry, and Behavior8955656220081832970110.1016/j.pbb.2008.02.009PMC2386143KarahanianEQuintanillaMETampierLEthanol as a prodrug: Brain metabolism of ethanol mediates its reinforcing effectsAlcoholism: Clinical and Experimental Research3560661220112133252910.1111/j.1530-0277.2011.01439.xPMC3142559McCance-KatzEFKostenTRJatlowPDisulfiram effects on acute cocaine administrationDrug and Alcohol Dependence5227391998978800310.1016/s0376-8716(98)00050-7McMahonFGDisulfiram-like reaction to a cephalosporinJAMA: Journal of the American Medical Association243239719806445429VasiliouVMalamasMMarselosMThe mechanism of alcohol intolerance produced by various therapeutic agentsActa Pharmacologica et Toxicologica (Copenhagen)583053101986294313310.1111/j.1600-0773.1986.tb00114.x

## Pharmacogenetic Studies for Improving Efficacy of Current Medications

Researchers have conducted pharmacogenetic trials for improving the efficacy of four drugs to treat AUD: naltrexone and acamprosate as well as two off-label medications, ondansetron and topiramate. Although gabapentin shows promise for reducing heavy drinking and increasing abstinence (Mason et al. 2012, 2014), to date no one has conducted pharmacogenetic trials on this drug. The pharmacogenetic studies of naltrexone, acamprosate, ondansetron, and topiramate are discussed at length below, and table 1 compares their effect sizes when studied using a pharmacogenetic approach and a nonpharmacogenetic approach.

### Naltrexone

The FDA approved oral naltrexone to treat AUD in 1994. It is relatively safe and well tolerated, with only a few reported nonspecific adverse effects (Chochrane 2010). At typical dosages, commercially available naltrexone, known as levo-naltrexone, primarily inhibits µ-opioid receptors (MOR) (Ziauddeen et al. 2013). The idea behind using naltrexone for AUD came from studies showing that some alcohol-dependent individuals have an endogenous opioid deficiency (Oslin et al. 2003). Both rodent models and human imaging studies show an acute increase in endogenous opioid released upon alcohol ingestion, which instigates its reinforcing effects (Gianoulakis 1996). Phase l/ll human laboratory trials conducted prior to FDA approval showed that naltrexone reduced alcohol cravings and reduced relapse to heavy drinking (O’Malley et al. 1992; Volpicelli et al. 1992).

The majority of clinical trials conducted in the United States to compare the efficacy of naltrexone to a placebo have shown that the drug is more effective in reducing drinking severity than promoting abstinence (Litten et al. 2013). In addition, although a recent multivariate meta-analysis of 41 single- and multisite pharmacotherapy trials conducted from 1992 to 2009 found that the effect size for naltrexone was modestly higher than placebo, its clinical success for promoting abstinence and reducing heavy drinking has declined steadily since the earliest single-site studies (Del Re et al. 2013). This failure of chronic treatment with naltrexone may, in part, be explained by the finding by Gelernter and colleagues (2007) that chronic exposure to opioid antagonists results in upregulation of cell-surface MOR density and function.

Studies into whether there are genetic markers that predict whether certain people respond better than others to naltrexone mostly have focused on a polymorphism of the *OPRM1* gene, which encodes for MOR subtype 1. The single nucleotide polymorphism (SNP), called rs1799971, is the most extensively studied *OPRM1* polymorphism in alcoholism research. It results from the substitution of an A nucleotide with a G nucleotide in exon 1 of *OPRM1* (Anton et al. 2008). The resulting allele is called A118G or Asn40Asp. The allelic differences are associated with both altered binding capacity and expression levels of MOR subtype 1 across species. Specifically, the G allele is associated with increased binding capacity for β-endorphin in cultured oocytes (Bond et al. 1998) and reduced mRNA and protein expression levels (Mague et al. 2009; Zhang et al. 2005), suggesting a relative baseline deficit of MOR subtype 1.

The first pharmacogenetic trial to study the use of naltrexone for treating AUD (Oslin et al. 2003) examined whether differences in rs1799971 influenced outcome. The retrospective, exploratory study used a double-blind, placebo-controlled 12-week treatment trial, with 141 alcohol-dependent individuals of European descent. The results indicated that people who carried at least one copy of the G allele and received naltrexone relapsed to heavy drinking at lower rates and took longer to do so than people who did not carry the G allele and received naltrexone. Although the results are intriguing, the study combined two disparate clinical trials and did not find a statistically significant interaction between naltrexone and the genotypes.

Since then, several other groups also have investigated whether rs1799971 affects drinking severity in naltrexone-treated individuals. By far the largest was conducted by Anton and colleagues (2008), using a subset of genetic samples from participants in the Combined Pharmacotherapies and Behavioral Interventions for Alcohol Dependence (COMBINE) study. The researchers analyzed the effectiveness of naltrexone in 604 Caucasians recruited at 11 academic sites across the United States. All of the study participants met criteria for DSM–IV alcohol dependence upon entering the study and were recently abstinent. The study found that participants who carried the rs1799971:G allele who also received naltrexone had fewer days of heavy drinking, defined as more than five standard drinks for men and four standard drinks for women, after 16 weeks of treatment. Interestingly, the G-allele carriers in the group that received only naltrexone without cognitive behavioral treatment had significantly more abstinent days during the 16-week treatment period (*P* = 0.01 to 0.03) than all other genotype-by-treatment groups.

Along with the traditional measures of treatment effectiveness, the authors constructed an outcome measure called “rates of good clinical outcome.” They defined this as the following:

“abstinent or moderate drinking without problems, a maximum of 11 (women) or 14 (men) drinks per week, with no more than 2 days on which more than 3 drinks (women) or 4 drinks (men) were consumed, and 3 or fewer alcohol-related problems endorsed on the Drinker Inventory of Consequences scale during the last 8 weeks of treatment”

(Anton et al. 2008, p. 138).

The authors found that the naltrexone-treated G-allele carriers were more than five times more likely to have rates of good clinical outcomes than all other treatment-by-genotype groups combined. The fact that all significant findings were present only in those who did not receive psychotherapy in addition to naltrexone—a finding which the authors explained as the pharmacological effects of naltrexone that were not masked by psychotherapy—has attracted caution in interpreting these findings.

Results from numerous subsequent studies have failed to replicate the predictive ability of rs1799971 (Arias et al. 2014; Coller et al. 2011; Foulds et al. 2015; Oslin et al. 2015) for improving naltrexone treatment response. Negative findings in these smaller statistically underpowered studies likely indicate that rs1799971 has a modest effect on naltrexone’s effectiveness. Given that rs1799971 alleles are more prevalent in Caucasian and Asian populations (table 2), naltrexone likely would be most beneficial in these populations (Ray et al. 2012). Supporting this argument, a few human laboratory trials have demonstrated that both European and Asian male and female heavy social drinkers carrying rs1799971:G, who were treated with naltrexone, had reduced craving for alcohol compared with people who received the placebo (Ray et al. 2010, 2012).

### Acamprosate

The FDA approved acamprosate to treat AUD in 2004, but it mainly is used in Europe for maintaining abstinence presumably by reducing craving, especially after alcohol detoxification (Cochrane 2011). Contrary to these findings, some studies suggest that acamprosate prevents relapse, not through altered craving (Umhau et al. 2011) but rather by reducing central nervous system hyperexcitabilty (Dahchour et al. 1998) and by causing a negative affective state during alcohol withdrawal (Cole et al. 2000).

Acamprosate consists of two acetylhomotaurine molecules linked by a calcium salt (Kalk and Lingford-Hughes 2014) with a chemical structure similar to the amino acid neurotransmitters gamma-aminobutyric acid (GABA), glutamate, glycine, aspartate, and taurine. It is thought that acamprosate stabilizes the chemical balance in the brain that would be disrupted by alcohol withdrawal. However, the molecular mechanisms involved are unclear. Many studies have shown that acamprosate has dose-dependent agonistic effects at GABA_A_ receptors and weak antagonistic effects at N-methyl-d-aspartate (NMDA) receptors and metabotropic glutamate receptor 5 (mGLuR5) (Krystal et al. 2006; Pierrefiche et al. 2004). A more recent study by Spanagel and colleagues (2014) showed that acamprosate’s antirelapse effects are, in fact, exerted via calcium that is incorporated in its formulation, rather than through effects of acetylhomotaurine on GABA and glutamate receptors (Spanagel et al. 2014).

Researchers working in European populations have found a few genetic polymorphisms that predict treatment response to acamprosate. Ooteman and colleagues (2009), for example, examined a polymorphism found in a GABA_A_ receptor gene called *GABRB2*. They found that alcohol-dependent patients carrying the TT genotype of the *GABRB2* C1412T polymorphism had reduced physiological responses (measured by decreased heart rate) to alcohol cues than patients carrying the C allele (Ooteman et al. 2009). Another study examined a polymorphism associated with a gene called *GATA4*, which encodes a transcription factor for atrial natriuretic peptide and has been associated with alcohol addiction (Kiefer et al. 2011). The SNP, called rs13273672, has an A allele and a G allele. The study found that study participants who carried the A allele had improved abstinence levels after 90 days of acamprosate treatment compared with patients carrying the G allele (Kiefer et al. 2011). This study also showed that patients carrying two copies of the A allele had increased plasma levels of atrial natriuretic peptide, providing a biological mechanism for the statistical association with treatment outcome. In another study, Spanagel and colleagues (2005) examined polymorphisms in a gene called *Per2,* which is associated with circadian cycles. The researchers demonstrated that mice with a mutation in *Per2,* known as *Per2^Brdm1^* mutant mice, reduced their drinking following acamprosate treatment (Spanagel et al. 2005). Additional biochemical examination showed that the *Per2^Brdm1^* mutant mice had a deletion in the PAS domain of the Per2 protein that resulted in reduced glutamate transporter Eaat1 expression levels and in turn increased synaptic glutamate levels. The same study examined alcohol intake in a population of Caucasian individuals treated with acamprosate and found that those who carried a protective allele located within a regulatory region of *PER2* intron 3 had lower alcohol intake (less than 300 g/day) than those who did not carry the allele. These findings need to be replicated in independent studies to validate their pharmacogenetic relevance in acamprosate treatment.

### Ondansetron

The FDA has approved ondansetron to treat postoperative and chemotherapy-induced nausea. The drug attaches to a number of receptors, dampening their ability to respond. It shows a low affinity to 5-HT_1A_, 5-HT_2_, adrenergic α1 and α2, dopamine receptor subtype 2, muscarinic M2, µ opioid receptor, benzodiazepine, and histamine H_1_ receptors. But it has a much larger affinity for 5-HT_3_ receptors, which have been associated with alcohol consumption. 5-HT_3_ receptors are ligand-gated ion channels that mediate the fast depolarization of neurons. They regulate dopamine release and are located densely in the brain’s mesocorticolimbic region. Alcohol stimulates 5-HT_3_, enhancing dopamine release and thereby increasing the risk of alcohol misuse. Selectively blocking 5-HT_3_ receptors attenuates dopamine release. Indeed, two studies showed that, in mice, alcohol intake had an inverse relationship with the expression levels of 5-HT_3_ receptors in the amygdala (Ciccocioppo et al. 1998; Hensler et al. 2004). Further characterizing the relationship between alcohol drinking and 5-HT_3_ receptors, Hodge and colleagues (2004) demonstrated that drinking behavior in mice is mediated specifically by the 5-HT_3A_ subunit of the 5-HT_3_ receptor complex. Another study found that mice with high compulsive-like alcohol-seeking behavior had lower levels of CpG methylation in the promoter region of the *HTR3A* gene, which codes for 5-HT_3A_ (Barker et al. 2014). These mice required higher doses of ondansetron to reduce their compulsive-like alcohol-seeking behavior, suggesting that higher expression levels of the 5-HT_3A_ subunit are associated with compulsive alcohol-seeking tendencies.

Findings from rodent models (Kostowski et al. 1994; Meert 1993) and subsequent human laboratory studies conducted with alcoholic individuals showed that ondansetron was able to reduce drinking (Sellers et al. 1994). One study suggested that it reduced drinking only in people with a biological predisposition to develop alcoholism before age 25 (Johnson et al. 2000). By testing a 16-fold dose range, this study also found that the most effective dose (4 µg/kg of body weight) is about 1,000 times smaller than the commercially available form for its FDA-approved indication. Independent replication studies have not all found the same link between age of onset and ondansetron’s treatment efficacy (Kranzler et al. 2003), and even Johnson and colleagues (2011) failed to find a significant effect of ondansetron, combined with cognitive–behavioral therapy, in reducing drinking among early-onset alcoholics (Johnson et al. 2011).

To examine whether certain subgroups respond better to ondansetron, Johnson and colleagues (2011) tested ondansetron in two subgroups of alcoholics based on their genotype for the serotonin transporter gene *SLC6A4*-promoter region functional polymorphism 5-HTTLPR (L/S). They found that patients treated with ondansetron who carried the LL genotype (5-HTTLPR:LL) drank about 1.5 fewer standard drinks on a drinking day and had 10 percent more abstinent days, compared with all other treatment by genotype groups (Johnson et al. 2011). A unique strength of this pharmacogenetic study was that the researchers randomly assigned participants to receive treatment (ondansetron plus CBT or placebo plus CBT) based on their 5-HTTLPR genotypes, which provided ample statistical power to detect the genetic effects. The researchers also further refined the 5-HTTLPR:LL group by adding another functional polymorphism in *SLC6A4* (SNP rs1042173[T/G]) that researchers had shown alters mRNA expression levels (Seneviratne et al. 2009). Adding this refinement markedly increased patients’ response to ondansetron: Carriers of both 5-HTTLPR:LL and rs1042173:TT genotypes who received ondansetron drank about 2.6 fewer drinks on a drinking day, and the percentage of abstinent days within the 3-month treatment period increased to 15.5 percent, compared with all other treatment by genotype groups. Only a small human laboratory trial has been reported so far to support the findings of the above pharmacogenetic trial. In a human laboratory trial, Kenna and colleagues (2014) demonstrated that alcohol-dependent individuals with 5-HTTLPR:LL genotype significantly reduced their alcohol consumption in response to 0.5 mg/day ondansetron treatment both in a naturalistic and a human laboratory environment under a self-administration model.

Johnson and colleagues (2011) selected the two SLC6A4 functional polymorphisms to personalize ondansetron in the above-mentioned study, because the serotonin transporter is the main modulator of serotonergic signaling. However, ondansetron does not bind to the serotonin transporter. Its primary target is the 5-HT_3_ receptor and, more specifically, the 5-HT_3A_ subunit. When a serotonin molecule binds to a 5-HT_3A_ subunit, a signal is propagated along the postsynaptic neuron, and this signal is blocked by ondansetron. The 5-HT_3A_ subunits heteromerize with 5-HT_3B_ subunits to form functionally efficient 5-HT_3_ receptors. Hence, in a secondary analysis, Johnson and colleagues (2013) re-analyzed the sample from their 2011 study to include polymorphisms from the two genes encoding 5-HT_3A/B_ subunits—*HTR3A* and *HTR3B* (Johnson et al. 2013). They found that genotypes across *HTR3A* and *HTR3B* were better able to identify subgroups of alcoholic individuals who would respond to ondansetron treatment compared with the two *SLC6A4* polymorphisms. The specific *HTR3A* and *HTR3B* predictive genotypes identified in this study were rs1150226–AG, rs1176713–GG and rs17614942–AC, respectively. When all individuals carrying any one or more of these three genotypes, along with the previously identified SLC6A4:LL/TT genotypes, were pooled together into one group, they predicted the number of drinks per drinking day, percentage of abstinent days, and heavy drinking days with larger effect sizes (table 1). The major drawback of this exploratory study is the small sample size. Large multisite randomized trials are needed to validate the findings of both pharmacogenetic trials by Johnson and colleagues (2011 and 2013).

### Topiramate

Researchers have tested topiramate as a promising agent to treat AUD in several clinical trials. Topiramate “decreases alcohol reinforcement and the propensity to drink (Johnson et al. 2007*a*, p. 4)” by facilitating GABA-A receptors and antagonizing AMPA and Kainate glutamate receptors (Angehagen et al. 2005; Braga et al. 2009; Poulsen et al. 2004; Simeone et al. 2011), which, in turn, reduce dopamine levels in mesocorticolimbic systems (Johnson et al. 2007*a*).

Johnson and colleagues (2003) conducted the first randomized, placebo-controlled trial (RCT) with topiramate for treating AUD. They tested a daily dose of up to 300 mg per day over a period of 12 weeks in a relatively small heterogeneous population of men and women. They reported a moderate to high effect size (0.7) for reducing heavy drinking by about three standard drinks on a drinking day, and a comparable effect size (0.76) for improving abstinence by about 27 percent. Follow-up studies (see table 1) carried out in larger populations with similar doses of topiramate have found it effective over a placebo, albeit with smaller effect sizes for reducing heavy drinking or improving abstinence (table 1). Several other RCTs also have reported no effect of topiramate on treating AUD (Kampman et al. 2013; Likhitsathian et al. 2013). A recent meta-analysis (Blodgett et al. 2014), performed with data from seven RCTs conducted between 2003 and 2014, supported a small to moderate effect for topiramate. Table 2 displays the effect sizes of topiramate on three drinking measures common to four of the seven RCTs. Although topiramate was reported to be more effective than other medications tested for AUD, higher rates of adverse effects observed in RCTs are a concern limiting its use. The most common adverse effects include cognitive dysfunction (Johnson et al. 2003), paresthesias (Kampman et al. 2013), and taste abnormalities (Johnson et al. 2008).

Pharmacogenetic tests of topiramate have focused on an SNP for a gene encoding one of topiramate’s primary receptor targets: the kainite Gluk1 receptor. The SNP, called rs2832407, is on a gene called *GRIK1*, and is an intronic substitution of nucleotides C-to-A. Kranzler and colleagues (2009) found that the minor allele A is associated with alcohol dependence. In a study examining whether the alleles influenced the effectiveness of topiramate, Ray and colleagues (2009) reported that patients treated with 300 mg of topiramate and who carry at least one copy of the A allele (AC or AA) had an increased risk for adverse events compared with patients with two copies of the C allele (CC). In an RCT that separated European participants by their genetic profile (CC, AA, or AC), Kranzler and colleagues (2014) compared the effectiveness of 200 mg topiramate with a placebo. They found that topiramate only decreased heavy drinking and increased abstinence rates in participants carrying two C alleles. For participants carrying AC or AA alleles, placebo and topiramate had similar effects on both drinking measures. If replicated in larger populations, this finding may facilitate the successful use of topiramate at a lower dose, reducing the adverse events that have restricted its use.

## Other Notable Genetic Polymorphisms

In recent years, researchers have compiled several large-scale genomic datasets that they have used to identify a handful of genetic variants that seem to influence the development of DSM-IV–defined alcohol dependence. Prospective clinical trials conducted in treatment-seeking populations will be critical to translating these findings into pharmacogenetics or improving medication efficacy and safety.

The strongest findings from genome-wide association studies (GWAS) to date are observed for the genes encoding the alcohol-metabolizing enzymes aldehyde dehydrogenase (ALDH) and alcohol dehydrogenase (ADH). Identifying genetic variations in these alcohol-metabolizing enzymes may have significant implications on pharmacological effects of some potential and currently used AUD medications. For example, oral naltrexone, ondansetron, sertraline, finasteride, and olanzapine all undergo significant first pass metabolism in the liver. Converging effects of these medications and genetic variations on alcohol metabolism should be considered as potential pharmacogenetic markers to personalize AUD treatment.

To date, the most consistently replicated polymorphism associated with alcohol metabolism is the SNP rs671 in the *ALDH2* gene, which is mapped to chromosome 12q24.2 and encodes the mitochondrial ALDH isozyme ALDH2. The SNP rs671 arises from a G to A allele transition. Researchers consistently have found that the rs671:A allele protects Asians against developing alcoholism (Tan et al. 2012) and is associated with slower metabolism of acetaldehyde, which leads to an aversive disulfiram-like reaction (Liu et al. 2005). The rs671:A allele is not reported in African and European populations (National Center for Biotechnology Information, 2015). However, researchers have found that another polymorphism, called ALDH1A1^*^2 in the ALDH1A1 gene, is associated with greater risk for alcoholism in African populations (Moore et al. 2007; Spence et al. 2003) but not in Asian populations (Otto et al. 2013).

Another SNP associated with alcoholism-related traits and gastro-intestinal tract cancers in Asian and European populations is called rs1229984 and is found in the *ADH1B* gene that encodes the β subunit of ADH1 (McKay et al. 2011; Park et al. 2013; Wu et al. 2013). It consists of an A allele and a G allele. Bosron and Li (1986) found that the A allele increases the capacity of ADH to oxidize alcohol into acetaldehyde by several-fold. A variant of one of the other two ADH1 subunits—the A allele of the *ADH1C* gene, called SNP rs698—also increases the capacity of ADH to oxidize alcohol into acetaldehyde by several-fold (Bosron and Li 1986).

## Characterizing Treatment Effects at the Molecular Level by Examining Gene Expression

Changes in drinking patterns or adverse events associated with a treatment, which typically are used to measure “treatment response” are, in fact, on some level determined by changes in the expression of multiple genes involved in drinking behavior that result from an extremely complex combination of environmental factors and the strength and duration of treatment.

Several studies have examined how alcohol alters gene expression patterns in postmortem humans, as well as in rodent brains and in vitro cell cultures. These studies have looked both at candidate genes and at a global genome-wide level. Especially with the advancement of gene expression technologies, new data have emerged not only on differentially expressed genes but also on underlying mechanisms of expression changes. Table 3 presents notable findings from human postmortem studies with potential pharmacogenetic implications that have not yet been investigated.

Studying gene expression mechanisms in living humans is understandably daunting. Nevertheless, exploring gene expression alterations is essential in clinical trials that aim to understand how medications work to change drinking behavior. In fact, researchers can examine gene expression using easily obtainable peripheral tissue, such as blood, combined with neuroimaging techniques to clarify how changes seen in the blood correlate with what is happening in the brain.

The clinical trial of odansetron by Johnson and colleagues (2011) reported preliminary data that shed light on their finding that patients carrying the SLC6A4:LL genotype responded better to ondansetron. Specifically, they found that study participants carrying the LL genotype were more likely than other participants to have an increase in SLC6A4 gene expression in blood cells (Seneviratne and Johnson 2012). As a future direction, it would be equally important to investigate the length of time the gene expressions persist during a medication-free follow-up period and whether the reversal of expression to premedication state would lead to relapse.

A newer technology for studying gene expression in living people is the creation of what is called induced pluripotent stem cells (iPSC) from tissues such as skin, allowing researchers to obtain cell cultures consisting of neurons and glia (Johnson et al. 2007*b*; Takahashi and Yamanaka 2006). The iPSC technology still is in its infancy, and only a few studies have used this relatively expensive technology in psychiatric research. In the drug addiction field, the only reported study to use iPSC-derived neural cells is a study by Lieberman and colleagues (2012) that examined the effects of alcohol on gene expression of NMDA receptors and their function. They found that expression levels of the NMDA receptor genes *GRIN1*, *GRIN2A*, and *GRIN2D* increased following cell cultures exposed to alcohol for 7 days. These findings corroborate earlier reports from human postmortem brain studies and findings from animal research and support using iPSC as a potential minimally invasive method to study molecular mechanisms in neurons. However, several challenges to this technology remain before it is ready for wider use in preclinical research, including high cost, inefficiency in producing mature cell types with realistic functionality, and difficulty developing cultures enriched with mature (desired) cells and without undifferentiated (undesired) cell types that retain the potential for tumor formation in vivo.

## Conclusion

In the past few years, many studies have focused on scrutinizing genetic polymorphisms that alter a person’s vulnerability to develop AUD. Association of these polymorphisms in shaping response to medications, or pharmacogenetics, only has begun recently. And although only a handful of published studies address AUD pharmacogenetics, those that have demonstrate a clear advantage over prescribing a common pill to all.

That said, several crucial steps are needed prior to applying these findings to clinical practice. First, the findings from published studies must be validated in larger, independent, preferably phase III, randomized placebo-controlled clinical trials. It also is widely accepted that the genetic architecture of different racial or ethnic groups tends to differ, although 99 percent of the human genome is shared among all races. This raises the possibility that what works for one ethnic population may not be optimal for another. The pharmacogenetic trials discussed above were conducted in predominantly Caucasian populations. Intriguingly, as shown in table 2, all of the genetic markers found to alter treatment efficacy of naltrexone, ondansetron, and topiramate in the few published phase II clinical trials show a significant variation in their prevalence among different racial groups. For example, pharmacogenetic markers for efficacy of ondansetron, which were found in a study of alcoholics of European descent (Johnson et al. 2011), are more prevalent in individuals with African ancestry and rare to nonexistent in East Asians. Furthermore, other genetic polymorphisms that modulate the function of the reported genetic markers also may vary among racial populations, rendering them inconsequential in nontested ethnic populations. Thus, only studies conducted in separate racial populations could decipher the clinical use of pharmacogenetic markers discovered in alcoholics of European ancestry.

Second, all pharmacogenetic trials conducted to date have used a candidate polymorphism approach. The tested genetic polymorphisms have proven to predict efficacy successfully over the conventional treatment approach. Nevertheless, much more comprehensive analyses are needed to explore the existence of other more predictive genetic markers of treatment efficacy. One approach would be to sequence the entirety of genes that include the selected polymorphisms. Another approach would be to conduct a GWAS with samples collected from a treatment trial, rather than a population-based genetic study, designed to detect genetic associations of disease vulnerability. This is especially important as treatment response and disease vulnerability may not necessarily share common polymorphic associations with the same magnitude of effects. Indeed, a GWAS analysis requires a large population of more than 1,000 participants completing the trial, which only can be obtained in multicenter trials. One solution to achieving such an ambitious task would be to require collection of genetic material, for future testing, from all National Institute on Alcohol Abuse and Alcoholism–funded AUD medication treatment trials. It also is important to note challenges with conducting prospective stratified studies. These studies are strengthened if researchers can study equal numbers of people with different versions of the marker under investigation. This design allows researchers to compare directly treatment response between groups carrying the marker and those not carrying the marker. However, it also is more challenging to enroll participants into genotype groups if the minor allele is rare in the population. Under such circumstances, a treatment by genotype group for people carrying a major allele would fill out much quicker than the treatment-by-genotype group for people carrying the minor allele. This could lead to potential selection bias, which is not a concern in randomized controlled clinical trials where the genetic samples are analyzed retrospectively.

Third, a genetic marker, predictive of greater treatment response or adverse events to a medication, ideally should be a surrogate for a pathophysiological process underlying AUD and/or a physiological alteration caused by the medication itself. Genetic markers detected in statistical association analyses should be tested for their functionality and response to both the medication and alcohol. This requires a more vigorous collaborative effort from clinical, translational, and basic science researchers. In addition to the scientific challenges, a number of practical issues such as privacy and confidentiality, provider training, and access to genetic testing facilities warrant consideration for the clinical application of pharmacogenetics. Despite these challenge, the pharmacogenetics approach is by far the most promising advancement in AUD treatment.

## Figures and Tables

**Table 1:** Effect Sizes in Pharmacogenetic and Nonpharmacogenetic Phase ll AUD Treatment Trials

	**Effect Size**	

**Medication and End-point Variable**	**Nonpharmacogenetic Trials**	**Pharmacogenetic Trials Effect Size (Gene Tested)**
**Naltrexone**		
Relapse to heavy drinking	0.247 (Del Re et al. 2013)	
Percent days abstinent	0.143 (Del Re et al. 2013)	
Good clinical outcome	Not measured	>0.8 in carriers of rs1799971:G allele (Anton et al. 2008)
**Ondansetron**		
Drinks per drinking day	NS; ondansetron vs. placebo main effects (Correa et al. 2013; Johnson et al. 2000, 2011)	0.87 in carriers of any one or more of the following genotypes → rs1150226:AG, rs1176713:GG, and rs17614942:AC; 0.59 when carriers of SLC6A4:LL and rs1042173: TT are added to the above group (Johnson et al. 2013)
% heavy drinking days	NS; ondansetron vs. placebo main effects (Correa et al. 2013; Johnson et al. 2000, 2011)	0.78 in carriers of any one or more of the following genotypes → rs1150226:AG, rs1176713:GG, and rs17614942:A; 0.42 when carriers of SLC6A4:LL and rs1042173: TT are added to the above group (Johnson et al. 2013)
% abstinent days	NS; ondansetron vs. placebo main effects (Correa et al. 2013; Johnson et al. 2000, 2011)	0.68 in carriers of any one or more of the following genotypes → rs1150226:AG, rs1176713:GG, and rs17614942:AC; 0.43 when carriers of SLC6A4:LL and rs1042173: TT are added to the above group (Johnson et al. 2013)
**Topiramate**		
Drinks per drinking day	0.45 (Johnson et al. 2003, 2007*a*; Rubio et al. 2009)	
% heavy drinking days	0.62 (Johnson et al. 2003, 2007*a*; Kranzler et al. 2014; Rubio et al. 2009)	Effective only in rs2832407:CC carriers but not in carriers of rs2832407:AC/AA (Kranzler et al. 2014)
% abstinent days	0.46 (Johnson et al. 2003, 2007*a*; Kranzler et al. 2014; Rubio et al. 2009)	Effective only in rs2832407:CC carriers but not in carriers of rs2832407:AC/AA (Kranzler et al. 2014)

All effect sizes are given in Cohen’s *d*. NS: Nonsignificant.

**Table 2:** Frequencies of Pharmacogenetic Markers in Ethnic/Racial Populations

		
**Medication**	**Pharmacogenetic Marker (Gene-Polymorphism: Genotype)**	**African**	**Caucasian**	**East Asian**	**South Asian**
**Naltrexone**	OPRM1-rs1799971:GG/GA	0.023	0.292	0.622	0.693
**Ondansetron**	HTR3A-rs1150226:AG	**0.470**	0.134	Fixed	0.023
	HTR3A-rs1176713:GG	**0.113**	0.088	0.048	0.136
	HTR3B-rs17614942:AC	0.077	**0.097**	Fixed	0.023
	SLC6A4-5HTTLPR:LL	**0.582**†	0.334††	0.109‡	0.191‡‡
	SLC6A4-rs1042173:TT	**0.736**	0.283	0.042	0.216
**Topiramate**	GRIK1-rs2832407:CC	0.019	**0.354**	0.205	0.273

All frequency data are from HapMap, unless specified otherwise. Highest population frequencies are in boldface letters.

† Douglas et al. 2011; Gelernter et al. 1998; Herman et al. 2011; Kraft et al. 2007; Roy et al. 2007.

†† Biederman et al. 2009; Douglas et al. 2011; Foley et al. 2004; Frisch et al. 1999; Geijer et al. 2000; Gerra et al. 2005; Gonda et al. 2010; Gokturk et al. 2008; Grabe et al. 2012*a*,*b*; Hallikainen et al. 1999; Herman et al. 2011; Illi et al. 2011; Iordanidou et al. 2010; Kronenberg et al. 2008; Landaas et al. 2010; Merenakk et al. 2011; Michaelovsky et al. 1999; Minelli et al. 2011; Mrazek et al. 2009; Mujakovic et al. 2011; Noskova et al. 2008; Pivac et al. 2009; Polito et al. 2011; Stoltenberg et al. 2012; van der Zwaluw et al. 2010; Volf et al. 2009.

‡ Choi et al. 2006; Chong et al. 2000; Chu et al. 2009; Gelernter et al. 1997; Hong et al. 2003; Katsuyama et al. 2008; Kim et al. 2006, 2007; Kweon et al. 2005; Li et al. 2007; Matsushita et al. 2001; Narita et al. 2001; Shin et al. 2010; Yamakawa et al. 2005; Yu et al. 2002.

‡‡ Banerjee et al. 2006; Guhathakurta et al. 2006; Vijayan et al. 2009, Kumar et al. 2007, 2012; Margoob et al. 2008; Sikander et al. 2009, Tibrewal et al. 2010.

**Table 3:** Potential Pharmacogenetic Targets Detected in Human Postmortem Brain Studies in Alcohol-Dependent Subjects and Animal Studies

**Potential Medication Targets**	**Altered Genes (Reference)**	

	**Human Postmortem Studies**	**Animal Studies**
Acamproate	↑NMDA subunit genes *GRIN2B* and *GRIN2D* in hippocampus (Enoch et al. 2014); ↓*GRIN2D* in the central amygdala (Jin et al. 2014); ↓*GRIN2A* in caudate n. (Bhandage et al. 2014)	↓*GRIN1* with chronic ethanol use in dorsolateral prefrontal cortex and ↑*GRIN1-1* isoform and ↓*GRIN1-2* isoform in OFC of male cynomolgus monkeys (Acosta et al. 2010).
Topiramate	↑*GRIA4* and *GRIK3* in hippocampus (Enoch et al. 2014); ↓*GRIA1*, *GRIA4*, *GRIK2*, and *GABRA2* in the central amygdala (Jin et al. 2014)	*GRIA2* flop mRNA levels in OFC and *GRIA3* flip and flop and *GRIA4* flop mRNAs in DLPFC positively correlated with daily ethanol intake in male cynomolgus monkeys (Acosta et al. 2011).
Ondansetron (for association with QT interval prolongation)/Topiramate	↓*SCN4B* in PFC (Farris et al. 2014)	↑*SCN4B* in limbic areas in mice (Mulligan et al. 2006, Tabakoff et al. 2008)
Ondansetron/SSRIs	↑*TPH2* expression in dorsal and median raphe nuclei (Bach et al. 2014)	
Baclofen	↓*GABBR1* in cortex through intron 4 alternative mRNA splicing (Lee et al. 2014) and hippocampus (Enoch et al. 2012)	↓*GABBR1* in hippocampus in P rats (Enoch et al. 2012)
Naltrexone	↑*PDYN* and *PDYN* in dorsolateral-PFC, *OPRK1* in OFC and *PDYN* in hippocampus (Bazov et al. 2013)	↓synaptosomal *OPRK1* receptor expression in mesolimbic brain regions (Nizhnikov et al. 2014) in Sprague-Dawley rats; ↑*POMC*, *PDYN* and *PENK* in nucleus accumbens in rats (Bordner and Deak 2015); ↑*PDYN* amygdala and nucleus accumbens in rats (D'Addario et al. 2013; Lam et al. 2008)
Canabinoid	↑*CNR1* in PFC of suicidal alcoholics (Erdozain et al. 2014)	↓*CNR1* in caudate-putamen, ventro-medial nucleus of the hypothalamus, hippocampus and ↑ in dentate gyrus (Ortiz et al. 2004); ↓*CNR1* in whole brain (Stringer et al. 2013)
Olanzapine	↓*DRD2* receptor protein levels in carriers of Taq1A polymorphism in the caudate nuclei (Noble et al. 1991)	↓*DRD2* in the nucleus accumbens and the hippocampus (Bice et al. 2008; Thanos et al. 2004)

↑ upregulated genes; ↓ downregulated genes; OFC—orbitofrontal cortex; DLPFC—dorsolateral prefrontal cortex; PFC—prefrontal cortex.

## Notes

Glossary

Allele: A fragment of DNA that can differ among individuals of the same species at a specific location of a chromosome. The difference can be just a single nucleotide or several nucleotides.
Exon: Genes are made up of segments of DNA called introns and exons, where the exon represents that part of the gene that is used to create the mature form of RNA, which is then translated into amino acids and make up a protein molecule.
Intron: Genes are made up of segments of DNA called introns and exons. The introns are parts of the DNA that are transcribed into the immature form of RNA but are spliced out before the RNA is translated into a protein.
Nucleotides: The subunits of nucleic acids, such as DNA and RNA, consisting of a nitrogen base (adenine [A], thymine [T], guanine [G], and cytosine [C]), a five-carbon sugar (deoxyribose or ribose, respectively), and at least one phosphate group.
Polymorphism: Differences in DNA sequences found within different individuals of the same species at the same location in the chromosome.
Single nucleotide polymorphism (SNP): A consistent change in a single nucleotide (A, T, C, or G) in a DNA sequence that can be found within members of a population at the same location in the chromosome. For example, the same fragment of DNA from two individuals may have the sequence, TCAGGT and TCAAGT, with a difference in a single nucleotide.
